# Transcriptome-based strategies for identifying aluminum tolerance genes in popcorn (*Zea mays* L. var. *everta*)

**DOI:** 10.1038/s41598-023-46810-9

**Published:** 2023-11-08

**Authors:** Vitor Batista Pinto, Pedro Marcus Pereira Vidigal, Maximiller Dal-Bianco, Fabricio Almeida-Silva, Thiago Motta Venancio, José Marcelo Soriano Viana

**Affiliations:** 1 Departamento de Biologia Geral, Universidade Federal de Viçosa (UFV), Viçosa, MG 36570-000 Brazil; 2 Laboratório de Biologia Celular e Tecidual, Centro de Biociências e Biotecnologia (CBB), Universidade Estadual do Norte Fluminense Darcy Ribeiro (UENF), Campos dos Goytacazes, RJ 28013-602 Brazil; 3 Núcleo de Análise de Biomoléculas (NuBioMol), Centro de Ciências Biológicas. UFV, Viçosa, MG 36570-000 Brazil; 4 Laboratório de Bioquímica Genética de Plantas/BIOAGRO. UFV, Viçosa, MG 36570-000 Brazil; 5 Laboratório de Química e Função de Proteínas e Peptídeos, CBB. UENF, Campos dos Goytacazes, RJ 28013-602 Brazil

**Keywords:** Abiotic, Plant breeding

## Abstract

Aluminum (Al) toxicity limits crop production worldwide. Although studies have identified genes associated with Al tolerance in crops, a large amount of data remains unexplored using other strategies. Here, we searched for single substitutions and InDels across differentially expressed genes (DEGs), linked DEGs to Al-tolerance QTLs reported in the literature for common maize, and investigated the alternative splicing regulated by Al^3+^ toxicity. We found 929 substitutions between DEGs in Al-tolerant and 464 in Al-sensitive inbred lines, of which 165 and 80 were non-synonymous, respectively. Only 12 NS variants had deleterious predicted effect on protein function in Al-tolerant and 13 in Al-sensitive. Moreover, 378 DEGs were mapped in Al-QTL regions for the Al-tolerant and 213 for the Al-sensitive. Furthermore, Al stress is primarily regulated at the transcriptional level in popcorn. Important genes identified, such as *HDT1, SWEET4a, GSTs, SAD9, PIP2-2, CASP-like 5*, and *AGP*, may benefit molecular assisted popcorn breeding or be useful in biotechnological approaches. These findings offer insights into the mechanisms of Al tolerance in popcorn and provide a ‘hypothesis-free’ strategy for identifying and prioritizing candidate genes that could be used to develop molecular markers or cultivars resilient to acidic soils.

## Introduction

Aluminum (Al) is one of the most abundant elements in the Earth’s crust. In acid soils, with pH values < 5, the phytotoxic species Al^3+^ is solubilized in soil solution and becomes one of the major problems of crop cultivation by interfering with root function and reducing plant development^1,2^. Al toxicity inhibits root growth and biomass, causes nutrient imbalance, changes in photosynthetic activities, increased oxidative stress and disturbances in carbon dioxide assimilation rates^3^.

At molecular level, Al-mediated signaling pathways involve various processes, including the perception and triggering of stress responses and resistance to Al in roots. These processes result from induced changes in cellular homeostasis, direct interaction between Al^3+^ and plasma membrane Al-receptors, and Al-induced inhibition of membrane transport and/or involvement of plasma membrane signal transducers. These alterations form the basis for signal transduction cascades that activate Al-resistance pathways: the transcriptional activation of biosynthetic enzymes and membrane transporters, which are central to exclusion and tolerance mechanisms; and the post-translational modifications activating and regulating protein functions^2^.

The well-known documented tolerance mechanisms of Al stress in higher plants includes exclusion and detoxification mechanisms. The exclusion mechanism consists of organic acid (OA) exudation, preventing the ion Al^3+^ from entering the root apex, forming the non-toxic complex Al-OA. On the other hand, the detoxification mechanism consists of neutralizing the toxic Al^3+^ absorbed through sequestration and detoxification in subcellular compartments and/or translocating it away from the root tip^2^.

Al tolerance is considered a quantitative trait in maize. Quantitative trait loci (QTL) mapping studies for Al tolerance in common maize identified genomic regions associated with this trait across several chromosomes^4–8^. Despite its agronomic importance, *ZmMATE1*^7^ and *ZmALMT1* and *ZmALMT2*^9,10^ have been characterized in common maize, but other genes and mechanisms involved in the tolerance to the phytotoxic Al ion are currently unknown.

Popcorn (*Zea mays* L. var. *everta*) has an important role in United States economy and also has a high demand in Brazil^11^. Like traditional maize, popcorn is extensively cultivated through tropics regions in Brazil, thus attracting the attention of breeders to obtain cultivars adapted to Brazilian conditions^12^. Al toxicity affects root development and causes damage and cell disorganization in the apical region in popcorn seedlings, ultimately compromising their growth and nutrient uptake^13^. The tolerance mechanisms in popcorn seedlings are involved with the increasing of sucrose content in the roots and shoots, starch decreasing in roots, and induced secretion of malate and fumarate^14^.

Gene expression is highly influenced by different environmental conditions and can trigger various point mutations across the genome. Plants exhibit diverse transcriptional, physiological, and fitness responses to abiotic stress^15–17^, and understanding how these variations affects gene expression as well as plant fitness and adaptation is crucial to providing solutions for crop breeding in the context of climate change. Beyond point mutations, alternative splicing is a dynamic post-transcriptional regulatory mechanism that produces multiple protein variants and regulates many physiological processes essential for plant growth and development, especially in response to stress conditions^18^. In this way, RNA-seq studies allow obtaining a large amount of data and provide new perspectives to uncover transcript dynamics and altered patterns under adverse environment conditions.

Recently, the gene expression profile of two popcorn inbred lines contrasting in Al-tolerance were tracked after 72 h of stress, revealing the mechanisms involved in a long-term Al-exposure^19^. This study used the RNA-seq approach and detected genes already known to be related to Al-tolerance, as well as others not previously described in hydroponic or soil experiments. Transcriptomic data analysis can provide valuable information on expressed genes in QTL regions, alternative splicing and SNPs associated with stress tolerance in plants. However, strategies to reduce the list of candidates are necessary when selecting candidates to use in breeding programs.

In this study, we performed comprehensive and extensive analyses to investigate potential candidates for Al-tolerance in popcorn. We assessed single substitutions and InDels across the differentially expressed genes (DEGs), linking them to Al-tolerance QTLs previously reported in the literature for common maize, and investigated the post-transcriptional regulation of popcorn under Al-stress. Our findings provide novel insights into the roles of different molecular processes underlying Al-tolerance in popcorn. They are useful to be explored in genetic engineering tools and/or as biomarkers in popcorn breeding programs.

## Results and discussion

### Al stress triggers polymorphisms in expressed genes

**Figure 1:**
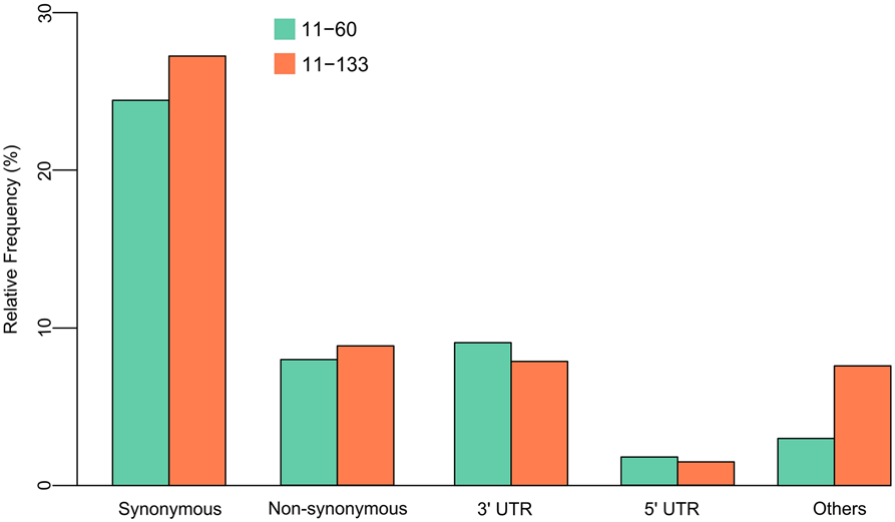
Polymorphisms among differentially expressed genes in Al-contrasting inbred lines under Al-stress.

A total of 929 substitutions were identified among 128 DEGs in the Al-tolerant inbred line (11–133), and 464 substitutions were found among 67 DEGs in the Al-sensitive inbred line (11–60) (Supplementary Table 1). In both inbred lines, most of the substitutions were synonymous, and variations were also found in untranslated regions (UTRs) (Fig. 1).

From the non-synonymous variants, a PROVEAN analysis showed that 12 of them presented deleterious predicted effect on protein function in the 11–133 (Table 1), and 13 in the 11–60 inbred line (Table 2). DEGs presenting deleterious predicted effects on protein function, either in 11–133 or in 11–60, are related to abiotic stress response, i.e. Glutathione transferase 7 (*GST7*, Zm00001d042104), Phenylalanine ammonia-lyase 1 (*PAL1*, Zm00001d033286), Histone deacetylase (*HDT1*, Zm00001d012092), and SNF1-related protein kinase regulatory subunit beta-1 (*SnRKβ1*, Zm00001d010662). Deleterious mutations can disable the biosynthesis of metabolites, altering plant responses to environmental stimuli^20^. However, under certain conditions, they can play a crucial role in adaptive evolution^21^. Otherwise, we detected 67 substitutions with neutral predicted effect (PROVEAN score < -2.5) on biological protein function in 11–133 (Supplementary Table 2) and 36 in 11–60 (Supplementary Table 3).

**Table 1:** Predicted deleterious variations in differentially expressed genes in the 11–133 Al-tolerant inbred line under Al stress.

Transcript	Description	Variant	PROVEAN score	Fold change
Zm00001d012092_T001	Histone deacetylase HDT1	254,P,T	− 3.577	2.00398
Zm00001d012221_T001	Stearoyl-acyl-carrier-protein desaturase 9	213,T,M	− 5.867	0.20829
Zm00001d021698_T001	Receptor-like protein kinase RK20-1	359,E,K	− 2.829	0.40400
Zm00001d021698_T001	Receptor-like protein kinase RK20-1	56,A,P	− 3.268	0.40400
Zm00001d026287_T005	UDP-N-acetylglucosamine diphosphorylase 2	261,G,E	− 5.537	2.11150
Zm00001d030915_T001	No annotated	66,N,T	− 3.111	0.17934
Zm00001d032857_T001	Protein kinase superfamily protein	332,D,N	− 2.524	0.48389
Zm00001d033286_T001	Phenylalanine ammonia-lyase 1	381,D,N	− 4.537	2.79448
Zm00001d034032_T001	Protein strictosidine synthase-like 3	162,G,S	− 5.333	2.06725
Zm00001d034077_T002	No annotated	125,R,C	− 8	2.35569
Zm00001d042104_T001	Glutathione transferase 7	24,R,Q	− 2.559	3.16789
Zm00001d046952_T001	Allantoate deiminase	225,A,G	− 3.268	0.46616

**Table 2:** Predicted deleterious variations in differentially expressed genes in the 11–60 Al-sensitive inbred line under Al stress.

Transcript	Description	Variant	PROVEAN score	Fold change
Zm00001d003757_T001	Bifunctional inhibitor/lipid-transfer protein/seed storage 2S albumin superfamily protein – DIR1	89,G,E	− 7	2.32511
Zm00001d010662_T001	SNF1-related protein kinase regulatory subunit beta-1	149,I,F	− 2.902	0.45543
Zm00001d012091_T001	Pro-resilin	112,G,A	− 2.6	0.25506
Zm00001d012091_T001	Pro-resilin	151,G,A	− 3.6	0.25506
Zm00001d016705_T002	ATPase inhibitor	36,R,W	− 8	0.34546
Zm00001d016724_T001	No annotated	26,A,S	− 3	2.37025
Zm00001d017746_T001	Tocopherol O-methyltransferase chloroplastic	172,A,G	− 2.52	2.48428
Zm00001d020696_T001	Asparate aminotransferase	418,A,V	− 2.57	0.35136
Zm00001d031971_T001	Vignain	185,A,T	− 2.506	3.52431
Zm00001d048065_T002	Tubulin alpha-1 chain	331,A,V	− 3.052	2.10818
Zm00001d049288_T001	No annotated	9,T,A	− 5	3.41702
Zm00001d049288_T001	No annotated	62,Q,L	− 7	3.41702
Zm00001d049288_T001	No annotated	84,H,Y	− 6	3.41702

Glutathione transferases are the main cellular detoxification enzymes that act against oxidative damage in plants^22^. The increased expression and activity of GST have been linked to aluminum tolerance in maize^23^. The predicted deleterious effect in *GST7* function was by a substitution of the basic Arg with Gly within the conserved N-terminal domain (Table 1). In GSTs, the N-terminal domain forms a thioredoxin-like fold, and changes in this domain have been correlated with high levels of GST expression in maize^24^. Additionally, a missense substitution with neutral predicted effect was detected in the C-terminal domain in *GST7* (Supplementary Table 2).

In addition to GSTs and the total glutathione accumulated in plant tissues under Al^3+^ toxicity, phenolic compounds are also regulated in response to this adverse condition, acting as potential defensive compounds. Phenylalanine ammonia-lyase is a key biosynthetic enzyme that produces intermediaries for polyphenolic compounds, and its activity increases in roots of Al-tolerant lettuce when exposed to Al toxicity^25^. In sorghum, the increasing of phytochelatins in roots can be related to Al^3+^ chelation^26^. Our findings suggest that this substitution in *GST7* and *PAL1* may confer an advantage in the detoxification of reactive oxygen species (ROS) and restriction of Al^3+^ in root cells of the Al-tolerant inbred line.

A single substitution in the polar residue region of *HDT1* resulted in the change of a Pro to a Thr (Table 1). Additionally, five substitutions with neutral predicted effects were found in *HDT1* (Supplementary Table 2). Histone acetylation and deacetylation play important roles in gene expression in eukaryotic cells. Maize exposed to different heavy metal stress conditions showed different acetylation levels due to different regulation of histone deacetylases^27^. These substitutions found in *HDT1* may be important for the adaptation of popcorn roots to Al toxicity, however, epigenetic regulation in maize under Al-stress is poorly understood and needs further investigation.

In the Al-sensitive inbred line, the down-regulated kinase *SnRKβ1* had a deleterious substitution in the AMPK1_CBM domain (Table 2). *SnRK1* plays several roles in abiotic stress resistance, and the decrease of its expression is associated with stress sensitivity^28^. This gene also regulates organic acid metabolism and other signaling pathways. SnRK1 knockdown lines of *Arabidopsis thaliana* under dark conditions altered the content of organic acids, revealing the importance of this gene in metabolic adaptation to stress^29^. Based on this, this amino acid change may cause unfavorable effects on protein function, increasing sensitiveness of 11–60 to Al^3+^.

Although this study focused on non-synonymous variations within protein-coding regions, silent substitutions and variations in intragenic regions among the DEGs may also have benefits for post-transcriptional mechanisms in response to Al-stress. While synonymous substitutions do not alter the amino acid sequence, they can still affect gene expression, protein folding, and the fitness of the organism^30^. Intragenic substitutions/InDels within 3′-UTR and 5′-UTR regions can alter gene expression by changing binding sites for transcription factors and miRNAs^20^.

A total of 164 variations were found in 3′-UTR and 5′-UTR regions in Al-tolerant inbred line, while 109 in the Al-sensitive, and none of these genes were shared in both inbred lines (Supplementary Table S1). The same transcript of *HDT1* that presented a NS variation (Zm00001d012092_T001) also presented two 3′-UTR variations, reinforcing its significance to Al-tolerance in popcorn. Furthermore, important up-regulated genes associated with abiotic stress presented variations in UTR regions. Were detected three variations in the 3′-UTR and 5′-UTR regions in *SWEET4a* (Zm00001d015905_T001), one in 3′-UTR in a Heavy metal transport/detoxification superfamily protein (Zm00001d027454_T001), one in 5′-UTR in *GST12* (Zm00001d027540_T002) and six in both UTR regions for *GST6* (Zm00001d027541_T001 and Zm00001d027541_T002). Curiously, *GST6* was detected as a central hub in in silico protein–protein network interacting with several Al tolerance related genes^19^.

These finds shed light for the importance of substitutions in UTR regions as an adaptative force to the plant deal with Al stress, enhancing the expression and impacting in the mRNA stability and translation efficiency of Al tolerance related genes.

### Linking RNA-seq data with QTLs previously described reduces the number of potential candidates for Al-tolerance

**Figure 2:**
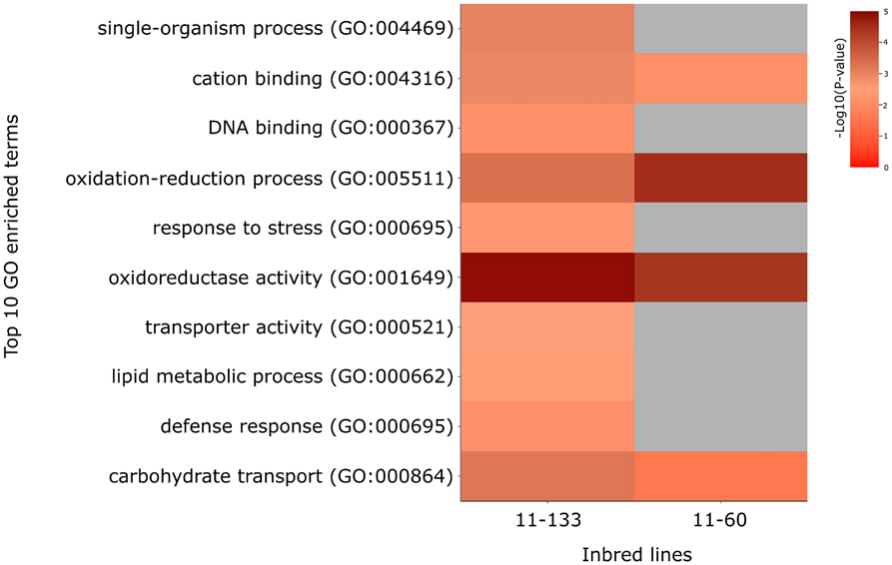
Gene ontology (GO) enrichment of top 10 GO terms of differentially expressed genes using PlantRegMap (http://plantregmap.gao-lab.org/) *Zea mays* database (*p-value* < 0.05).

**Figure 3:**
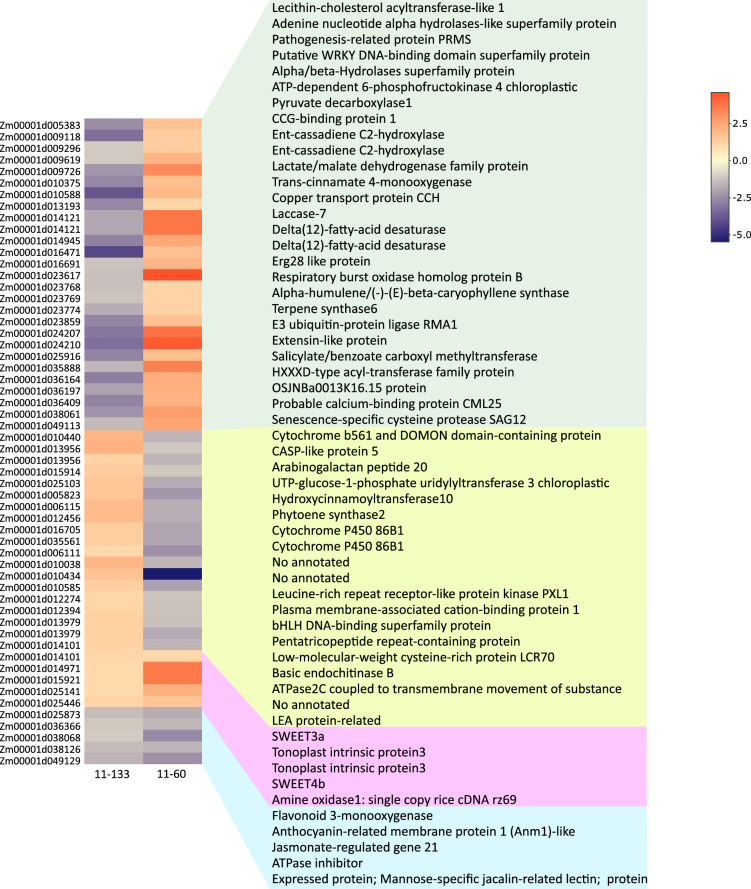
Heatmap of the unweighted pair group method (UPGMA) clustering of co-regulated genes between Al-tolerant and Al-sensitive inbred lines mapped within Al-related QTL regions in maize.

A total of 378 DEGs were identified within previous Al-QTL regions in 11–133 (Supplementary Table 4), and 213 genes in 11–60 (Supplementary Table 5). Gene ontology enrichment analysis showed that the majority of these DEGs were categorized under oxidoreductase activity and oxidation–reduction process, moreover, terms related to stress response and defense were enriched only in the Al-tolerant inbred line (Fig. 2). Out of this total, 56 DEGs were shared between both inbred lines (Fig. 3).

Al-related genes found in common maize, like *ZmMATE1*, *ZmALMT1* and *ZmALMT2*, were not differentially expressed in popcorn. This may be attributed to the timeframe of our study, which aimed to investigate the long-term molecular response to Al exposure, and to the genetic background of these popcorn inbred lines with the common maize genotypes tested in previous studies.

In 11–133, *HDT1* and Stearoyl-acyl-carrier-protein desaturase 9 (*SAD9*, Zm00001d012221) were mapped in *QTL7*^5^, and both showed changes with predicted deleterious effect on protein function, and the first transcript also presented variations in 3′-UTR region (Table 3, Supplementary Tables 1 and 4). *SAD* is a key enzyme in determining the global content of unsaturated fatty acids (UFA)^31^. Its expression decreased, and the predicted deleterious effect may lead to the reduction in the UFA content of lipids cells, changing lipid fluidity. Moreover, two Δ_12_-fatty-acid desaturases (Zm00001d023768 and Zm00001d023769) were down-regulated in 11–133 but up-regulated in 11–60 (Fig. 3, Supplementary Table 4), and mapped in *QTL2*. A high degree of fatty acid desaturation allows for high capability to stabilize the membrane fluidity, alleviating membrane damage from Al stress and limiting the entry of Al^3+^ into roots^32^.

**Table 3:** Al-tolerance QTLs identified in previous work using different genotypes of common maize (*Zea mays*).

ID	Chr	Markers	Population	Reference
*QTL1*	6	umc85/umc59	F2 generation derived by Cat-100–6 (Al tolerant) and S1587-17 (Al sensitive)	Sibov et al.^8^
*QTL2*	10	umc130/npi232		
*QTL3*	2	umc139/p-bnlg198	F3:4 families derived by L53 (Al sensitive) and L1327 (Al tolerant)	Ninamango-Cárdenas et al.^9^
*QTL4*	6	p-phi126/p-phi077		
*QTL5*	6	mmc0241/nc013		
*QTL6*	8	umc103/p-bnlg162		
*QTL7*	8	p-bnlg1031/p-phi080		
*QTL8*	4	umc1550	F2 generation derived by diallel crosses between L20, L08, and L06 (Al-sensitive) and L10 and L09 (Al-tolerant), and F2 derived by DKB747 (Al- tolerant) and DKB205 (Al-sensitive) commercial hybrids	Conceição et al.^10^
*QTL9*	5	bnlg1382		
*QTL10*	6	bnlg1154		
*QTL11*	8	umc1202		
*QTL12*	10	umc1318		
*QTL13*	6	p-phi126/umc1018	RILs derived by Cateto Al237 (Al-tolerant) and L53 (Al-sensitive)	Maron et al.^11^
*QTL14*	5	bnlg105/umc1019		
*QTL15*	2	S2_212940514	RILs derived by Cateto Al237 (Al-tolerant) and L53 (Al-sensitive)	Guimaraes et al.^12^
*QTL16*	3	S3_187460236		
*QTL17*	5	S5_30301926		
*QTL18*	6	ZmMATE1		
*QTL19*	8	S8_22681622		

Two aquaporins *PIP2-2* (Zm00001d005410 and Zm00001d014285) were detected in *QTL3, QTL14,* and *QTL17*^5,7,8^, and an ABC transporter (Zm00001d024600) in *QTL2*^4^ for the Al-tolerant inbred line (Supplementary Table 4). Aquaporins and ABC transporters play an essential role in the vacuolar sequestration of Al^3+^, promoting Al tolerance in plants^33^. Aquaporins *PIP1-1* and *PIP2* were recently identified in the maintenance of hydration in *Citrus limonia* L. plants when exposed to Al stress^34^. On the same way, ABC transporters facilitate vacuolar sequestration of Al, playing an important role in the tolerance mechanism^35^. Both are involved in diverse processes in plant growth and development under abiotic stress^36^.

The *SWEET3b* (Zm00001d023673) and *SWEET4a* (Zm00001d015905) were identified in *QTL2*^4^ and *QTL14*^7^ in 11–133 (Supplementary Table 4). In addition, *SWEET4a* presented variations in UTR regions (Supplementary Table 1). To deal with environmental stress conditions, plants need to maintain a strict regulation in the storage and transport of vacuolar sugar^37^. The SWEET genes play an important role in tolerance to osmotic stress, cold, high salinity, and drought^38^. The role of SWEET transporters is poorly understood in plants exposed to Al^3+^ toxicity, but these genes may be related to the Al tolerant phenotype in popcorn.

We identified DEGs involved in cell wall modification in 11–133 inbred line that were mapped across several Al tolerance QTL (Supplementary Table 4). These genes are mainly involved in primary cell wall and xyloglucan metabolism. ^39,40^ previously reported some of the same genes in Arabidopsis that are involved in Al tolerance. ^41^ detected a xyloglucan endotransglycosylase protein 8 precursor in the Al tolerance QTL *Alm1*. These findings suggest that the process of structural organization of cell wall is crucial for root growth and development under Al^3+^ toxicity conditions in the Al-tolerant inbred line.

CASP-like 5 (Zm00001d010038) and Arabinogalactan peptide 20 (*AGP*, Zm00001d010434) were both mapped in *QTL6*^5^ and found to be up-regulated in 11–133 but down-regulated in 11–60. CASP protein is involved in stress resistance and nutrient uptake^42^, and in Tamba black soybean under Al-stress, the expression of CASP protein is associated with root growth^43^. On the other hand, *AGP* plays multiple roles in plant growth and development, and under abiotic stress, it is involved in the thickening of cell wall by oxidative crosslinking mediating the stress signaling response^44^. The presence of these genes within an Al tolerance QTL may be responsive for the root and plant growth in acid soils.

In addition to reducing the number of candidates useful for developing molecular markers for marker-assisted selection or genomic selection for Al-tolerance, the identification of DEGs in QTL regions can provide insights into the molecular mechanisms underlying the traits controlled by these regions.

### Response to Al stress is primarily regulated at the transcriptional level

For the Al-tolerant inbred line, we observed a total of 25,026 RI; 15,357 A3SS; 11,782 A5SS; 10,786 SE; and 626 MXE events, respectively. A single gene (Zm00001d002141), which encodes a UBP1-associated protein 2A, underwent differential SE events, with three exons whose inclusions increased upon treatment. A similar frequency of AS events was observed for the Al-sensitive inbred line, which had 25,039 RI; 15,345 A3SS; 11,791 A5SS; 10,743 SE; and 618 MXE events, respectively. Three genes (Zm00001d047479, Zm00001d036136, Zm00001d044494) underwent differential SE events in the Al-sensitive inbred line. These genes encode a superoxide dismutase (*SOD*), CYSTM domain-containing protein, and pectin acetylesterase (*PAE*), respectively.

The small number of genes undergoing differential AS (DAS) reveals that post-transcriptional regulation does not play a significant role in popcorn response under long-term Al exposure. In 11–60, the three genes that underwent differential SE events are involved in abiotic stress response. The *SOD* is already known to play a role in the detoxification mechanism in maize under Al stress^45^. In Arabidopsis, CYSTM is involved in several developmental processes under abiotic stresses conditions^46^. Silencing *PAE* and annexin in hairy roots of *Medicago truncatula* increased the sensitivity to Al stress, suggesting that these gene may be involved in Al resistance response^47^. In rice, a significant change of AS profile was observed in the tolerant cultivar under Al stress^48^. Also in rice, the response to cadmium stress is highly controlled at the post-transcriptional level, as several differentials AS events were detected^49^. Similarly, rice response to alkalinity stress also triggered many differentials AS events^50^.

While a substantial number of DAS events were not associated with Al-tolerance in popcorn, we propose that post-transcriptional regulation plays a role in establishing timely patterns of downstream gene expression in response to stress. The interplay between transcriptional and post-transcriptional regulation, including AS, is highly complex and context-dependent, for these reasons we believe that post-transcriptional regulation may act during the growth and development of popcorn plants under Al-stress. Therefore, conducting additional analyses to explore the involvement of AS during a time course exposure of popcorn seedlings to Al may enhance our understanding of the complexity response to Al stress.

In this study, we have explored the transcriptomic data beyond gene expression and demonstrated the potential that this tool can offer to researchers in reducing targets and saving time for further analysis. This strategy has allowed us to identify several genes with a wide range of functions correlated with Al stress response, such transporters, enzymes involved in cell wall modification, fatty acid biosynthesis, and genes involved in stress response and plant development. Although searching for DEGs in Al tolerance QTL regions can significantly reduce the number of candidates to be explored, the genetic background differences between the genotypes used in QTL studies and our popcorn inbred lines may allow new regions that control Al-tolerance on unmapped chromosomes. Furthermore, as the Al stress response is majorly regulated at the transcriptional level, the genetic variations found in this study can be validated and functionally characterized to understand their roles in Al stress tolerance. This information can be used to develop molecular markers for Al tolerance, which can be utilized in breeding programs to develop cultivars that are resilient to the challenges posed by changing environmental conditions.

## Methods

### Plant material and selection of differentially expressed genes

The first step was the selection of the genes from our previous work^19^. The transcriptome was generated from seven years old seedlings of two contrasting inbred popcorn lines developed by the Popcorn Breeding Program of the Universidade Federal de Viçosa (Viçosa, Brazil): 11–133 (Al-resistant) and 11–60 (Al-sensitive). These genotypes were selected based on a previous study^13^ to screen inbred popcorn lines with different Al sensitivity. Seedlings with uniform growth were picked randomly and transferred to a nutritive solution^51,52^ with constant aeration to acclimate for 24 h. Then, the treatment group was subjected to aluminum stress with 540 µM of AlCl_3_ (160 μM Al^3+^) for 72 h. To assess only the Al effect on seedlings growth, the pH was adjusted to 4.5 for both control and treatment conditions and the seedlings maintained in a growth chamber at 25 °C with a 12/12 h light/dark cycle. RNA from roots was extracted and sequenced follow by gene expression analysis as described by Pinto et al. (2021)^19^.

### In silico mapping

To verify the localization of the DEGs within the previously described Al tolerance QTLs^4–8^, the positions of each delimiting QTL marker identified in different segregating populations in common maize (*Zea mays*) were examined using the MaizeGDB database (Table 3). To identify the DEGs of interest identified in our transcriptomic analysis inside the QTLs, we performed a simple comparison between the positions of the genes and the markers on the chromosome, as described in Mattiello et al.^41^.

### Polymorphism discovery and protein sequence variation prediction

The maize reference genome (B73.RefGen_v4) was downloaded from Phytozome database (https://phytozome.jgi.doe.gov). The reads were mapped to the reference genome using BWA-MEM algorithm of BWA version 0.7.17 (http://bio-bwa.sourceforge.net/)^53^. A flag was added to identify the respective popcorn sample in each mapping file. The mapping files were processed using SortSam, MarkDuplicates and BuildBamIndex tools of Picard version 2.18.27 (https://github.com/broadinstitute/picard/). Variants were called using FreeBayes version 1.2.0 (https://github.com/ekg/freebayes)^54^ with a minimum mapping quality of 20, minimum base quality of 20, and minimum coverage of 20 reads at every position in the reference genome. After variant calling, SNPs were filtered using vcftools version 0.16.15 (https://vcftools.github.io/index.html) and annotated using Variant Effect Predictor^55^ available on Ensembl Plants web server (http://plants.ensembl.org/Tools/VEP). The translated protein sequences were the analyzed using the PROVEAN (Protein Variation Effect Analyzer) software tool (http://provean.jcvi.org/index.php) to predict whether an amino acid substitution or InDel has an impact on the biological function of a protein. A PROVEAN score less than or equal to -2.5 was considered to have a significant biological impact on the protein function.

### Differential splicing analysis

The filtered reads were aligned to the maize reference genome (B73.RefGen_v4) using STAR^56^ with default settings. Replicate Multivariate Analysis of Transcript Splicing (rMATS)^57^ was employed to identify differential splicing events between the treatment and control conditions for both inbred lines. rMATS classifies alternative splicing (AS) into five categories, including skipped exon (SE), retained intron (RI), mutually exclusive exons (MXE), alternative 5′ splice sites (A5SS), and alternative 3′ splice sites (A3SS). To be considered for analysis, splicing events had to have at least 10 reads supporting exon inclusion (IJC + SJC) in all replicates. Differential splicing was considered statistically significant if the false discovery rate (FDR) was less than 0.05.

## Consent to participate

All authors consented to participate of this research.

## Declaration of use of plant material

The popcorn seeds used in this article followed the national standards required by Ministry of Agriculture, Livestock and Supply (MAPA), agency that regulates production, processing, repackaging, storage, analysis or seed trading activities in Brazil, according to Decree Nº. 10.586, of December 18, 2020, which regulates Law Nº. 10.711, of August 5, 2003. We emphasize that none of the seeds were collected for this work, once they belong to the Germplasm Bank of UFV and come from several cycles of interpopulation recurrent selection and more recently have been evaluated for some abiotic stresses by the Popcorn Breeding Program of UFV and that all works have the institution's full consent for its realization.

### Supplementary Information

Supplementary Table S1.

Supplementary Table S2.

Supplementary Table S3.

Supplementary Table S4.

Supplementary Table S5.

## Data Availability

The data sets supporting the results of this article are available in the NCBI SRA repository, http://www.ncbi.nlm.nih.gov/bioproject/508768.
